# STEAP2 promotes osteosarcoma progression by inducing epithelial–mesenchymal transition via the PI3K/AKT/mTOR signaling pathway and is regulated by EFEMP2

**DOI:** 10.1080/15384047.2022.2136465

**Published:** 2022-10-31

**Authors:** Dong Zhang, Haitao Liu, Weihua Wang, Gang Xu, Chenxiao Yin, Songgang Wang

**Affiliations:** a Department of Orthopedics, Suzhou Hospital of Anhui Medical University, Suzhou, P.R. China; b Department of Orthopedics, Xiangcheng No. 2 People’s Hospital, Suzhou, P.R. China; c Department of Orthopedics, Qilu Hospital, Cheeloo College of Medicine, Shandong University, Jinan, China

**Keywords:** STEAP2, osteosarcoma, PI3K/AKT/mTOR signaling pathway, EMT, invasion, metastasis

## Abstract

This study was designed to explore the prognostic significance and functionality of STEAP2 (six-transmembrane epithelial antigen of prostate 2) in osteosarcomas and determine whether EFEMP2 (Epidermal growth factor-containing fibulin-like extracellular matrix protein 2) targets STEAP2 to facilitate osteosarcoma cell infiltration and migration. STEAP2 expression in peritumoral tissues, osteosarcoma, benign fibrous dysplasia, osteosarcoma cells, normal osteoblastic hFOB cells, and various invasive subclones was evaluated using IHC, ICC, and qRT-PCR. We also evaluated the association between STEAP2 expression and disease outcome using Kaplan–Meier analyses and then investigated STEAP2 regulation and its functional effects using both *in vitro* and *in vivo* assays. The results revealed that the upregulation of STEAP2 in osteosarcoma tissues positively correlated with both the malignant osteosarcoma phenotype and poor patient outcomes. In addition, STEAP2 expression induced epithelial–mesenchymal transition (EMT) via the PI3K/AKT/mTOR axis and facilitated osteosarcoma cell infiltration and migration. Changes in EFEMP2 expression resulted in correlating changes in STEAP2 expression, with EFEMP2-overexpressing osteosarcoma cells exhibiting a less invasive phenotype and reduced EMT following STEAP2 inhibition. It is also worth noting that although EFEMP2 overexpression activated the PI3K/AKT/mTOR pathway promoting EMT, it did not affect osteosarcoma cells in which STEAP2 or Akt was knocked down. Thus, we can conclude that STEAP2 acts as an oncogene in osteosarcoma progression, while EFEMP2 enables PI3K/AKT/mTOR axis initiation and EMT by partly targeting STEAP2, thereby facilitating osteosarcoma cell infiltration and migration.

## Introduction

Osteosarcoma is a frequently occurring bone tissue malignancy, with a particularly high incidence rate in children and adolescents, with >75% of these cases recorded in patients aged 15–25 years. Osteosarcoma is characterized by early onset, easy metastasis, high malignancy levels, and fast growth,^1^ and despite traditional interventions such as surgical resection with chemotherapy, the five-year survival for these patients remains low at ˂70%. This is compounded by a ˂20% long-run survival rate among populations with metastatic and recurrent osteosarcoma, making the diagnosis devastating for most patients and their families.^2,3^ Unfortunately, there have been no studies describing any improved therapeutic options, which means that understanding the gene- and molecular-level regulation of osteosarcoma pathogenesis is critical to improving patient outcomes.

STEAP (six-transmembrane epithelial antigen of the prostate) is a broad-spectrum antigen known to function as key biomarker in prostate cancer. This protein, which was discovered in 1999 in an animal model of transplanted prostate cancer, is primarily expressed at the prostatic secretory epithelial cell–cell junction and named for its six potential transmembrane regions.^4^ Considering that the transmembrane region is flanked by hydrophilic amino and carboxyl terminals, STEAP may support a set of functions similar to those of ion channel and transporter proteins.^5^ The STEAP protein family consists of five members, namely, STEAP1, STEAP1b, STEAP2, STEAP3, and STEAP4. Both STEAP1 and STEAP2 are located on chromosome 7q21.13 and transcribed in the same direction, which may explain their co-expression in various cancer cells. In addition, these two proteins can form heterotrimers when they are co expressed,^6,7^ and recent studies have identified STEAP1 as an ideal target for broad-spectrum cellular and antibody immunotherapy. STEAP1 can also be widely used in clinical diagnosis and cancer treatment.^8–10^ However, studies on the relationship between STEAP2 and cancer have primarily focused on prostate cancer and remain fairly limited.^8^ STEAP2, with its location within the trans-Golgi network, is often also referred to as STAMP1 (six-transmembrane protein of prostate 1) and is known to cross the plasma membrane, suggesting that it may play a role in both secretory and endocytic pathways.^11–13^ Increased STEAP2 expression is thought to be a critical step in improving the invasive activity of prostatic oncocytes,^14^ while STEAP3, which is also known as TSAP6 (tumor suppressor activated pathway 6), is involved in the occurrence and development of various tumors through its functions in iron metabolism.^15–17^ STEAP4 was initially identified as part of the gene network regulated by androgen in prostate cancer cells and was first described as a metalloreductase. STEAP4 plays an important role in various metabolic disorders and tumorigenesis.^18–20^ However, the relationship between these STEAP family proteins and osteosarcoma is yet to be reported.

EFEMP2 is necessary for the formation of elastic fibers and the development of connective tissues but is also known for its dual effects of tumor inhibition and promotion, with these outcomes largely depending on tumor tissue type and microenvironment.^21–23^ In our previous study we reported that EFEMP2 facilitated epithelial–mesenchymal transition (EMT) and osteosarcoma development via its initiation of the PI3K pathway.^24^ We then went on to use an EMT PCR array to identify the impact of EFEMP2 inhibition on 90 EMT-related genes, which in turn identified STEAP1 and STEAP2 as the most impacted. Given the relationship between the STEAP family of proteins and tumor progression, we wanted to clarify whether these changes in EFEMP2 affect other STEAP proteins. qRT-PCR and western blot analyses confirmed that STEAP1 and STEAP2 expression was downregulated in response to a reduction in EFEMP2 level and upregulated in response to an increase in this protein level, but the expression of STEAP3 and STEAP4 remained unchanged in both scenarios (Supplementary Fig 1). Given these outcomes, this study mainly evaluated the roles of STEAP2 in osteosarcoma, its value as biomarker, and its specific interaction with EFEMP2, to better understand osteosarcoma pathogenesis.

## Materials and methods

### Specimen collection

We selected 150 cases of osteosarcoma, 60 cases of paratumoral tissue (1–2 cm outside the lesion) and 80 cases of bone fibrous dysplasia, from Qilu Hospital Pathology Department. No radio- or chemotherapy was implemented in any of these patients prior to surgery, and routine follow-up was completed. All of our protocols were approved by the Shandong University Qilu Hospital’s Medical Ethics Committee (Grant no. KYLL-2020-427), and all sample collection was only completed following our receipt of written informed consent from the patients or their families.

### Routine cell culture

Human osteosarcoma (MG63, U-2OS) and normal osteoblastic (hFOB) cells were procured from the CAS Cell Bank, and subclones MG63-1 and MG63-31, with high and low invasiveness, respectively, were isolated from our original MG63 cell line using single-cell cloning technology.^24^ All cell cultures were performed under aseptic conditions, and cells were grown in complete medium composed of 90% DMEM/F12 and 10% fetal bovine serum (Gibco), supplemented with double antibodies to prevent contamination, at a concentration of 1%. Cells were then cultivated in a 37°C incubator maintained at 5% CO_2_, and follow-up experiments were conducted when cell confluence reached 80%.

### Streptavidin-peroxidase (SP) immunohistochemical and immunocytochemical staining

Paraffin tissue sections were prepared by placing the sliced tissues in an oven at 65°C for 1 h to remove the paraffin wax, placing in dewaxing xylene for 15 min (this step was repeated twice), and immersing in anhydrous ethanol at 90% and 75%. These hydrated slides were then placed in 500 mL of citrate buffer (0.01 M) for high pressure antigen repair prior to their downstream application. Cell culture slides were prepared using logarithmic phase cells inoculated on coverslips and conventionally cultured for 24 h which were then rinsed in PBS and fixed in 4% paraformaldehyde for 30 min. We then used the following SP staining procedure on both our tissue and cell culture slides as per the kit (ZSGB-BIO) guidelines. The staining status of each sample was examined using an optical microscope, and three fields of view were randomly selected for scoring and rating, with each of these outcomes evaluated according to the intensity of the staining and the ratio of positive cells per field. Staining intensity was scored in 0–3 points as follows: completely negative (0), light brown (1), brown (2), and dark brown (3). Positive cells were then evaluated and used to create a positive cell ratio score as follows: 0, 0%; 1, 0–25%; 2, 25–75%, and 3, ≥75%. The total expression score ranged from 0 to 6, with low expression scoring ˂3 and high expression scoring ≥3. Each set of slides were scored by two pathologists, and discrepancies were evaluated and resolved via consultation.

### RNA extraction, reverse transcription, and qRT-PCR

Logarithmic cells were harvested into Eppendorf (EP) tubes and mixed with 1 mL of RNAiso PLUS (TaKaRa Biotechnology, China) to fully lyse the cells for total RNA extraction. RNA density and purity were then evaluated using a microplate reader and used as template for reverse transcription. All primers including those for housekeeping (β-actin) and target genes were designed and produced by TaKaRa Bioengineering Co., LTD., and are summarized in Table 1. PCR amplifications used 20 µL reactions composed of the qPCR reagent and cDNA templates and were performed on an ABI Prism SDS 7000 detector (Applied Biosystems Inc). The Ct values of each sample were calculated, and data were evaluated using the Light Cycler 480 System. All samples were quantified relative to the control, and the EMT PCR Array (Shanghai WcGene Biotech) assay was used to evaluate the responses of the EMT-associated genes in response to EFEMP2 downregulation.

**Table 1.** The sequence of primer in qRT-PCR.

Primer name	Sequences
STEAP1	F: 5ʹ‐ACAAGTTGCTAAACTGGGCATATCA‐3ʹ R: 5ʹ‐CAGTATTGCCAATCCCACAATTC‐3ʹ
STEAP2	F:5’-CGCTATGGTCCATGTTGCCTA-3’ R:5’-CCAAGGCTCATTATGCCAAAG-3’
STEAP3	F:5′-TGCAAACTCGCTCAACTGGAG-3′ R:5′-GAAGGTGGGAGGCAGGTAGAA-3′
STEAP4	F:5′-GGCTTTGGGAATACTTGG-3′ R:5′-GGACTGGACAAATCGGAAC-3′
CDH1	F:5’-GGATTGCAAATTCCTGCCATTC-3’ R:5’-AACGTTGTCCCGGGTGTCA-3’
CDH2	F:5’-CGAATGGATGAAAGACCCATCC-3’ R:5’-GCCACTGCCTTCATAGTCAAACACT-3’
VIM	F:5’-AACCTGGCCGAGGACATCA-3’ R:5’-TCAAGGTCAAGACGTGCCAGA-3’
SNAIL	F:5’-GCTCCCTCTTCCTCTCCATACC-3’ R: 5’-AAGTCCTGTGGGGCTGATGT-3’
SLUG	F: 5’-GAAGCATTTCAACGCCTCCAA-3’ R: 5’-GTTGTGGTATGACAGGCATGGAGTA-3’
TWIST	F: 5’-CAGCTACGCCTTCTCGGTCT-3’ R: 5’-CTGTCCATTTTCTCCTTCTCTGG-3’
ACTB	F: 5’-TGGCACCCAGCACAATGAA-3’ R: 5’-CTAAGTCATAGTCCGCCTAGAAGCA-3’

### Protein extraction and western blot

Experimental cells were harvested and then mixed with RIPA:PMSF (100:1) to facilitate total protein extraction, and protein concentration was then determined using a bicinchoninic acid kit (BOSTER). Equal concentrations (40 μg) of total protein were then separated by electrophoresis on a 10% separation gel and transferred to a polyvinylidene fluoride membrane. This membrane was then blocked for 1 h using nonfat milk (5%) and incubated overnight with a working solution of the relevant primary antibodies (EFEMP2 12004-1-AP, STEAP1 20199-1-AP, STEAP2 20201-1-AP, Twist 25465-1-AP, Proteintech; STEAP3 PA5-115969, STEAP4 PA5-115971, Invitrogen™; EMT Antibody Sampler Kit #9782, Cell Signaling Technology; PI3K ab86714, p-PI3K ab182651, AKT ab8805, p-AKT 38449, mTOR ab32028, p-mTOR ab109268, Abcam) at 4°C. The membranes were then washed using Tris-buffered saline with Tween® 20 and incubated with the secondary antibody for 1 h at room temperature. Membranes were then washed again and visualized using an enhanced chemiluminescent substrate kit (Millipore) and Image J software.

### Lentivirus transfection, RNA interference, and overexpression assays

LV-STEAP2-RNAi and LV-STEAP2-overexpressing virus were procured from Shanghai Genechem Co., Ltd and applied to increase or decrease their target gene levels. One virus was used to knockdown STEAP2 expression in the osteosarcoma subclone MG63-1 and the other to increase its expression in MG63-31 to produce the STEAP2-shRNA and EX-STEAP2 cell lines described in this study. Cells were seeded at 3000–5000 target cells per well in a 96-well microplate and incubated for 24 h prior to infection. These cells were then infected using a multiplicity of infection (MOI) of 50, with the infection volume determined as follows: viral volume = (MOI × cell counts)/viral titer. The cells were then cultured with the virus for 12 h before the media were replaced and cultured for an additional 72 h. Successful infections were identified by an approximately 80% transfection efficiency, and cells were routinely cultured for subsequent experiments. The down- and upregulation of STEAP2 was then verified by qRT-PCR, western blot, and ICC.

### Transwell chamber invasion/migration assays

Matrigel is a soluble matrix membrane component, known to be liquid at 4°C and polymerized at room temperature allowing it to be used to create bioactive gels that mimic the basement membrane. These attributes can then be exploited to evaluate cell invasiveness. Here, Matrigel was diluted in complete medium (dilution ratio 1:5), and 50 µL of the solutions was plated in the upper compartment filter membrane of the transwell chamber and mixed with 200 µL of cells (approximately 2 × 10^5^ cells). These plates were then grown under serum-free cultivation for 24 h before the media were removed and the lower chamber was filled with 600 μL of chemokine enriched media. The plates were then incubated at 37°C in 5% CO_2_ for 24 h before being fixed for evaluation. First the upper chamber was swabbed to remove any non-migrating cells, and then the filters and lower chamber were fixed in 4% paraformaldehyde for 30 min before staining with crystal violet for another 15 min. The chamber membranes were then washed, dried, cut, and sealed using neutral balsam with the bottom facing up. Five randomly selected fields were then evaluated using a microscope, and the number of cells crossing the membrane was counted. Transwell migration assays were then completed in an almost identical manner, except that the upper chamber was not covered in Matrigel. Each of these experiments were completed in triplicate.

### Cell proliferation assay using Cell Counting Kit-8 (CCK-8)

The differences in cell proliferation before and after transfection were assessed using a CCK8 kit (Beyotime Institute of Biotechnology, China). Briefly, 2000 cells/well were inoculated into a 96-well microplate, and eight wells were treated with 10 µL of CCK8 buffer every 24 h over 4 days. Two hours after the addition of the CCK8 buffer, all wells were evaluated using a microplate reader set to 450 nm with each of the values of the 4 days plotted to create the comparative proliferation curves used in our investigation. All of these evaluations were also completed in triplicate.

### Cell plate clone assay

Cells were seeded in 6-well microplates (1000 cells/well) and incubated at 37°C for approximately 14 days with media supplementation as necessary. Following this, the media were removed and the wells were fixed in 4% paraformaldehyde before staining with crystal violet. The stained plates were then placed on a white background, and the number of cellular colonies were enumerated. These experiments were replicated three times for each group, and the number and size of the colonies in each group were compared.

### Nude mice tumors

Twenty-five 3–4-week-old SPF BALB/C-nu/nu nude mice, with no differences in activity status and body weight, were randomly divided into five groups: MG63-1, MG63-1 STEAP2-shRNA1, STEAP2-shRNA2, MG63-31, and MG63-31 EX-STEAP2 groups, with five mice in each group. After harvesting approximately 1.0 × 10^7^ healthy cells per group, the cells were suspended in sterile saline (150 µL) and subcutaneously injected into the necks and backs of each animal. These animals were then fed normally, and tumors were evaluated using Vernier calipers every week. The data were then used to determine the tumor volume as follows: tumor volume = length × width^2^ × 0.5. Tumor growth was then described as a time (abscissa)-dependent graph of tumor volume (ordinate). All murine experiments were approved by the Shandong University Qilu Hospital’s Institutional Animal Care and Use Committee and completed following the principles and guidelines outlined in the Declaration of Helsinki and the Regulations on the Control of Laboratory Animals acts.

### Statistical analysis

Every experiment was performed in at least triplicate, and all groups were properly replicated. Count data were expressed as a ratio or constituent ratio, and measurement data were displayed as means ± standard deviations. Experimental data analysis was completed in SPSS 26.0, and pairwise comparisons of independent samples were completed using the Wilcoxon rank sum test. All multiple comparisons between independent samples were evaluated using the Kruskal–Wallis test, and inter-rate comparisons were completed using the Chi-square test. Finally, Kaplan–Meier and log-rank tests were used to complete the survival assessment of patients with cervical cancer, and GraphPad Prism 8.0.2 was used to visualize the data. Differences were considered significant when *p* < .05.

## Results

### Changes in EFEMP2 expression directly affect STEAP2 expression

The influence of EFEMP2 expression on EMT was assessed using an EMT PCR array that compared gene expression profiles before and after treatment with EFEMP2 targeting RNA interference. The assays revealed that these disruptions impacted not only the expression of several critical EMT effectors (e.g., N-cadherin, E-cadherin and vimentin) but also the expression of several other regulatory transcripts such as FGFBP1, MST1R, and WNT11, which were all upregulated in response to reduced EFEMP2, and VCAN, EGFR, ITGAV, ITGB1, MAP1B, COL5A2, DSC2, STEAP1 and STEAP2, which were all downregulated in response to reduced EFEMP2 (Supplementary Fig 1A). In addition, because the STEAP family is known to play a critical role in the tumor progression of various cancers, we went on to evaluate whether changes in EFEMP2 affected the expression of the STEAP proteins. qRT-PCR (Supplementary Fig 1B) revealed that STEAP1 and STEAP2 expression are downregulated in response to EFEMP2 knockdown, while their expression increased when EFEMP2 was overexpressed. Western blot (Supplementary Fig. 1C and D) further verified these changes at the protein level, confirming that EFEMP2 inhibition decreases the expression of STEAP1 and STEAP2, and EFEMP2 overexpression promotes STEAP1 and STEAP2 expression. Interestingly, both western blot and qRT-PCR confirmed that both STEAP3 and STEAP4 are EFEMP2 independent. Given that the relationship between STEAP proteins and osteosarcoma remains largely unknown, we used the data to develop a set of experiments designed to determine whether STEAP2 promotes the development of osteosarcoma and whether these functions are linked to EFEMP2 activity in these cells. The relationship between STEAP1 and osteosarcoma will be discussed in other study.

### STEAP2 expression in osteosarcoma, osteofibrous dysplasia, and paratumoral tissues

**Figure 1.**
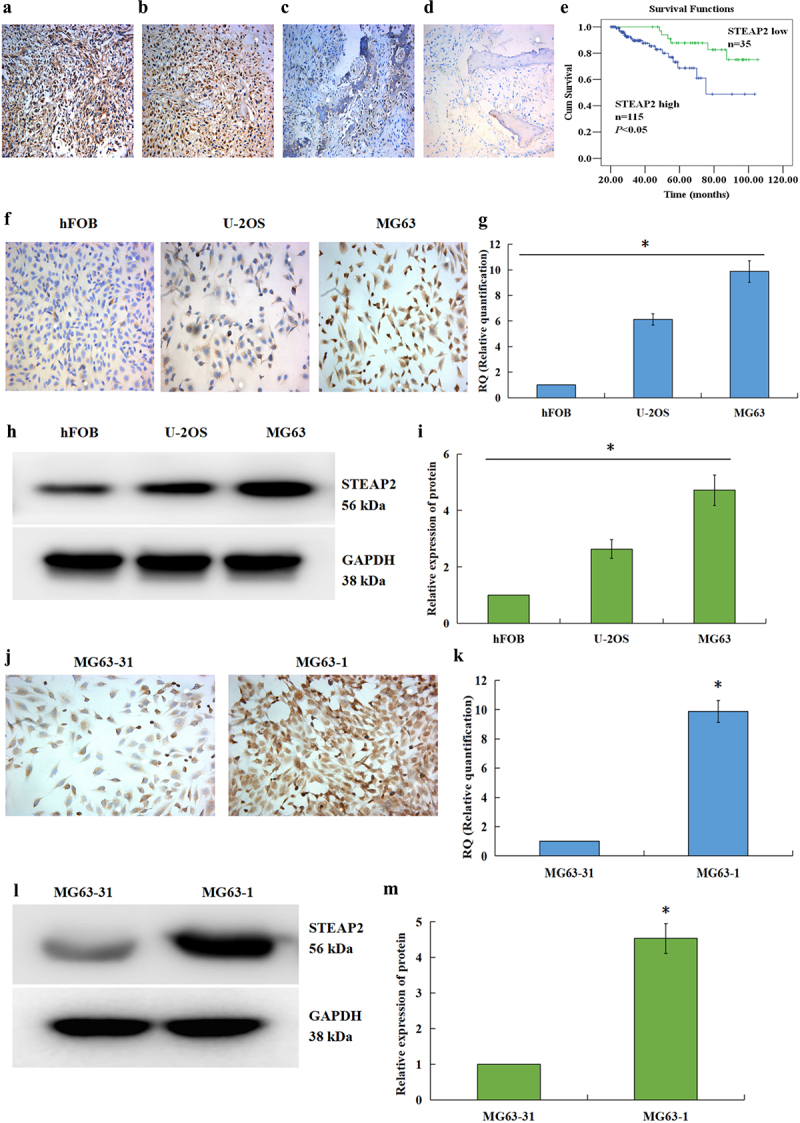
Expression of STEAP2 in osteosarcoma, osteofibrous dysplasia, paratumoral tissues, osteosarcoma cell lines, normal osteoblast cell line, and subclone cells with different invasiveness. The STEAP2 levels in osteosarcoma (AB), osteofibrous dysplasia (c), and paratumoral tissues (d) were determined with immunohistochemical assay. (e) According to Kaplan-Meier and Log Rank assessments, the outcome of osteosarcoma patients expressing STEAP2 protein lowly (green line) was prominently better than those expressing STEAP2 protein highly (blue line). By ICC (f), qRT-PCR (g), as well as western-blot (h) and its relative gray value (i), the STEAP2 mRNA and protein levels were estimated in osteosarcoma (U-2OS and MG63) and normal osteoblastic (hFOB) cells. Besides, the STEAP2 levels in the high invasive MG63-1 and the lowly invasive MG63-31 subclones were also determined with ICC (j), qRT-PCR (k), as well as western-blot (l) and its relative gray value (m). Magnification×200, *P < .05.

STEAP2 expression in osteosarcoma, osteofibrous dysplasia, and paratumoral tissues was evaluated using IHC, which revealed that STEAP2 was overexpressed in most osteosarcoma tissues (Figure 1 a and b) but showed a low expression in osteofibrous dysplasia (Figure 1c) and paratumoral tissues (Figure 1d). The increased expression was largely localized to the cytoplasm of the osteosarcoma cells, and STEAP2 expression displayed little variation across the various pathological subgroups of this disease. However, increased expression of STEAP2 positively correlated with lymph node metastasis, distance metastasis and poor differentiation (Table 2) in patients with osteosarcoma. Subsequent survival analysis also confirmed a strong positive correlation between STEAP2 and poor prognosis (Figure 1e).

**Table 2.** Protein expression of STEAP2 in human osteosarcoma tissues.

	N	STEAP2 low (-/+)		STEAP2 high (++/+++)		*X^2^*	*P*
		n	%	n	%		
Normal tissue	60	53	88.3%	7	11.7%	95.9	<0.01
Fibrous dysplasia	80	59	73.8%	21	26.2%		
Osteosarcoma	150	35	23.3%	115	76.7%		
Pathological type						3.0	>0.05
*Fibroblastic osteosarcoma*	54	9	16.7%	45	83.3%		
*Osteoblastic osteosarcoma*	52	16	30.8%	36	69.2%		
*Chondroblastic osteosarcoma*	44	10	22.7%	34	77.3%		
Cell differentiation						14.3	<0.01
*High and intermediate*	78	28	35.9%	50	64.1%		
*Low*	72	7	9.7%	65	90.3%		
Nodal status						26.9	<0.01
*Positive*	83	6	7.2%	77	92.8%		
*Negative*	67	29	43.3%	38	56.7%		
Distant metastases						18.2	<0.01
*Positive*	64	4	6.2%	60	93.8%		
*Negative*	86	31	36.0%	55	64.0%

### STEAP2 expression in osteosarcoma cell lines, normal osteoblasts, and subclone lines with different degrees of invasiveness

Given the above outcomes, we further evaluated STEAP2 expression *in vitro* using ICC (figure 1f), qRT-PCR (Figure 1g), and western blot (Figure 1 h and i). These assays revealed STEAP2 mRNA and protein expression in both osteosarcoma (U-2OS and MG63) and normal osteoblastic (hFOB) cell lines, with increased expression in the U-2OS and MG63 cell lines. We also reported that STEAP2 was differentially expressed in the highly invasive (MG63-1) and less invasive (MG63-31) subclone lines, with significant differences in its expression levels identified when comparing these lines to each other and the control (Figure 1j-m). These observations were then confirmed when comparing the IHC results of clinical tissue samples and both experiments revealed that STEAP2 expression increased in more invasive osteosarcoma cells. These observations led us to develop additional assays designed to probe deeper into the role of STEAP2 in osteosarcoma infiltration and metastasis. In these experiments we used RNAi to reduce STEAP2 expression in the MG63-1 subclone line and protein overexpression to increase STEAP2 expression in the MG63-31 subclone line. These alterations in turn allowed us to determine the effects of STEAP2 on invasiveness both *in vitro* and *in vivo*.

### Confirming changes in STEAP2 expression in response to RNAi or overexpression cassettes

**Figure 2.**
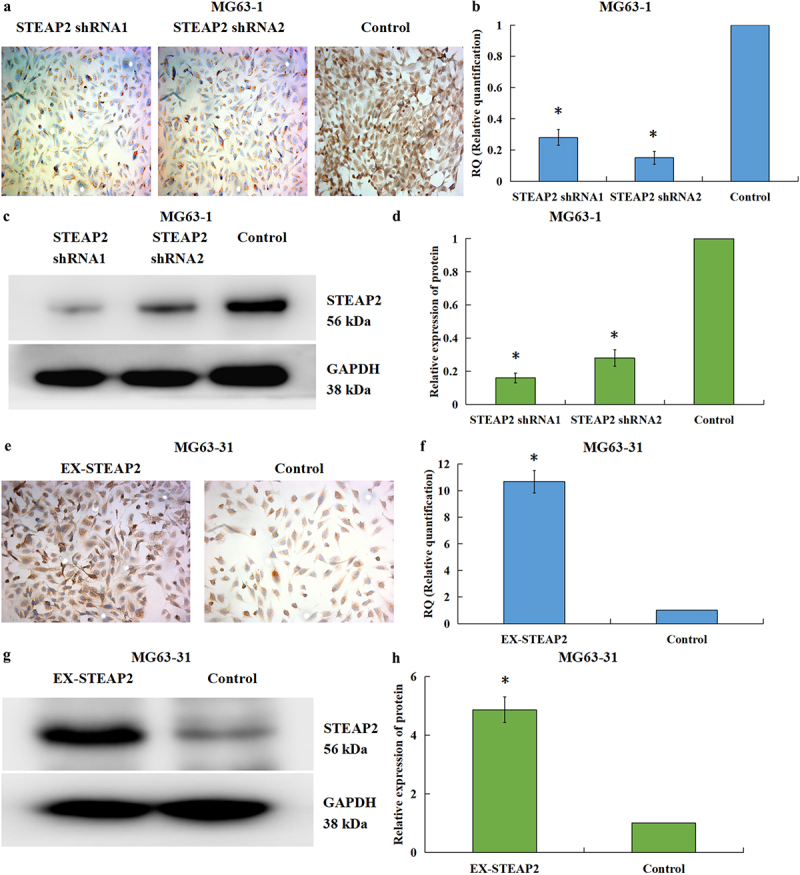
Identification of down-regulated or up-regulated STEAP2 expression in lentivirus knockdown or over-expression transfection systems. By ICC (a), qRT-PCR (b), as well as western-blot (c) and its relative gray value (d), the STEAP2 mRNA and protein expressions in MG63-1-shRNA1 and MG63-1-shRNA2 cells significantly decreased in contrast to those in the negative controls. Moreover, through a combination of ICC (e), qRT-PCR (f), as well as western-blot (g) and its relative gray value (h), the STEAP2 mRNA and protein expressions in MG63-31-EX-STEAP2 and control cells were also measured to verify the effect of over-expression transfection. Magnification×200, *P < .05.

MG63-1 and MG63-31 cells were transfected with STEAP2 shRNA1 and shRNA2 and EX-STEAP2 lentivirus. Using ICC (Figure 2a), qRT-PCR (Figure 2b), and western blot (Figure 2 c and d), downregulation of STEAP2 transcription was confirmed in both the MG63-1-shRNA1 and MG63-1-shRNA2 cell lines. Increased STEAP2 mRNA and protein expression profiles were then confirmed in MG63-31-EX-STEAP2 cell line using ICC (Figure 2e), qRT-PCR (figure 2f), and western blot (Figure 2 g and h). Taken together these evaluations confirmed that these novel subclone lines retained the expression profile of interest.

### Effects of changes in STEAP2 expression on the proliferation, clonogenicity, migration, and invasion of osteosarcoma cells

**Figure 3.**
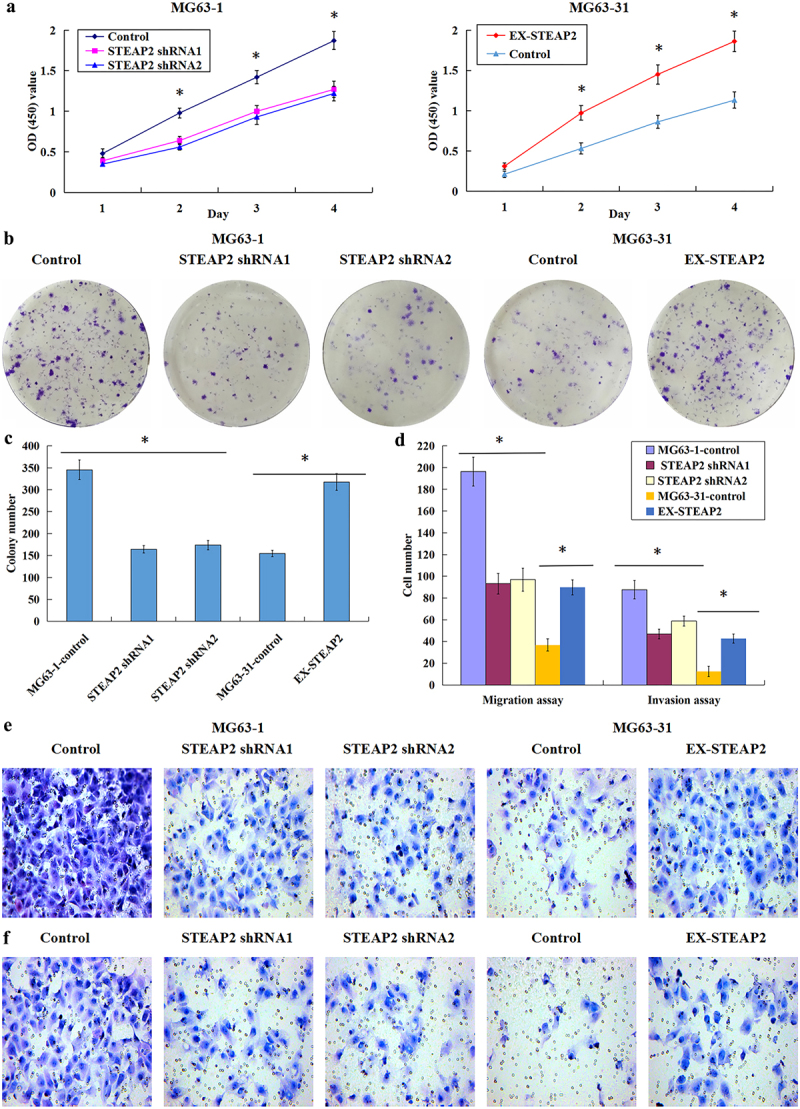
Effects of down-regulated or over-expressed STEAP2 on the proliferation, clonogenicity, migration, and invasion of osteosarcoma cells. (a) The down-regulation of STEAP2 significantly inhibited the MG63-1 subclone multiplication, whereas the STEAP2 up-regulation facilitated the MG63-31 subclone multiplication prominently. (b) The images of the plate clone formation experiment, the efficiency of colony formation of STEAP2-silenced cells reduced; however, the up-regulation of STEAP2 improved the colony formation efficiency of lowly invasive subclone. (c) The colonies formed in the STEAP2 shRNA1 and shRNA2 groups was lower in quantity pronouncedly than those in the negative controls, while the clones formed in the STEAP2 cDNA group were markedly increased in quantity compared to negative control group. (d) STEAP2 knockdown inhibited the infiltration and migration of osteosarcoma cells, whereas the STEAP2 over-expression facilitated such cellular events. The images of cell migration (e) and invasion (f) experiments, a smaller number of osteosarcoma cells transfected with STEAP2 shRNA1 and shRNA2 invaded through Matrigel or migrated through the PVDF membrane in contrast to the negative control cells. Moreover, the invading or migrating cells with up-regulated STEAP2 expression was pronouncedly higher in average number compared to the negative control cells. Magnification×200, *P < .05.

The downregulation of STEAP2 significantly inhibited cellular proliferation in the highly invasive MG63-1 cell line, while the upregulation of STEAP2 promotes proliferation in the less invasive cell line, MG63-31 (Figure 3a). This was further supported by the colony forming assays, which revealed that STEAP2-silenced cells produced fewer colonies than the control, while upregulation of this protein level increased colony formation in the low-invasive clone (Figure 3 b and c). Quantitative comparisons of cellular migration and invasion revealed that STEAP2 knockdown inhibits osteosarcoma cell infiltration and migration, while its overexpression increases both infiltration and migration (Figure 3d). The micrographs taken for both the migration (Figure 3e) and invasion (figure 3f) assays revealed a clear reduction in the number of invasive cells in the STEAP2 shRNA1/shRNA2 group when compared to that in the control. Moreover, the exact opposite was recorded in the overexpression group, with increases in STEAP2 producing concomitant increases in invasion and migration. Thus, we were able to conclude that STEAP2 inhibition is likely to reduce the proliferation, clonogenicity, infiltration, and migration of osteosarcoma cells, while its overexpression is likely to facilitate all of these outcomes.

### Effects of differentially regulating STEAP2 expression on tumor growth in a xenograft model

**Figure 4.**
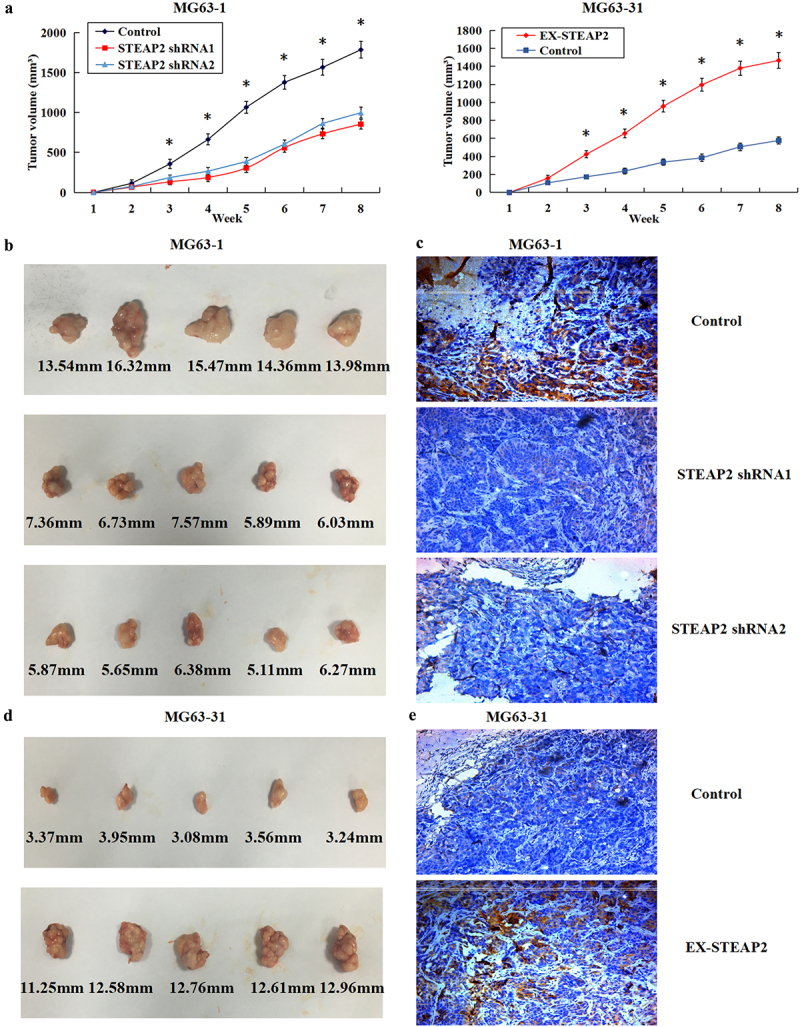
Effects of down-regulated or over-expressed STEAP2 on tumor growth in a xenograft model. (a) After eight weeks’ observation, tumor growth and size reduced in the STEAP2 shRNA1 and shRNA2-transfected groups, and in contrast, STEAP2 over-expression increased the rate and volume of subcutaneous tumor growth. (b) The STEAP2-shRNA-infected groups exhibited smaller mean tumor volume compared to the MG63-1 negative control. (c) By immunohistochemistry, the RNAi group exhibited lower STEAP2 level compared to the control group. (d) Compared to the control group, the mean tumor size of cells transfected with STEAP2 cDNA was much larger. (e) Immunohistochemistry revealed higher STEAP2 level in the STEAP2 over-expression group in contrast to the control group. Magnification×200, *P < .05.

The *in vivo* role(s) of STEAP2 were further investigated using a xenograft model. Here, 25 nude mice were inoculated with MG63-1 cells transfected with STEAP2 shRNA1 and 2 or STEAP2 cDNA-transfected MG63-31 cells and corresponding negative controls, respectively. There were 5 nude mice in each group. The mice were then monitored for 8 weeks, and tumor growth and size were compared between the groups. These comparisons revealed a clear decrease in tumor size and growth in the STEAP2 shRNA1 and shRNA2-transfected groups when compared to those in the control and a significant increase in both of these parameters in the STEAP2-overexpressing groups (Figure 4a). The STEAP2-shRNA-infected groups also exhibited smaller mean tumor volume than the MG63-1 control (Figure 4b), and IHC evaluations confirmed lower STEAP2 expression in the RNAi group (Figure 4c). Thus, we can confirm that the downregulation of STEAP2 expression inhibits tumorigenesis and evolution. By contrast, the mean tumor size of cells transfected with STEAP2 cDNA was much larger than that in the control group (Figure 4d) suggesting that increased STEAP2 expression (Figure 4e) likely promotes tumor growth *in vivo*.

### Effect of differentially expressing STEAP2 on key EMT hallmarks and the PI3K/Akt/mTOR pathway

**Figure 5.**
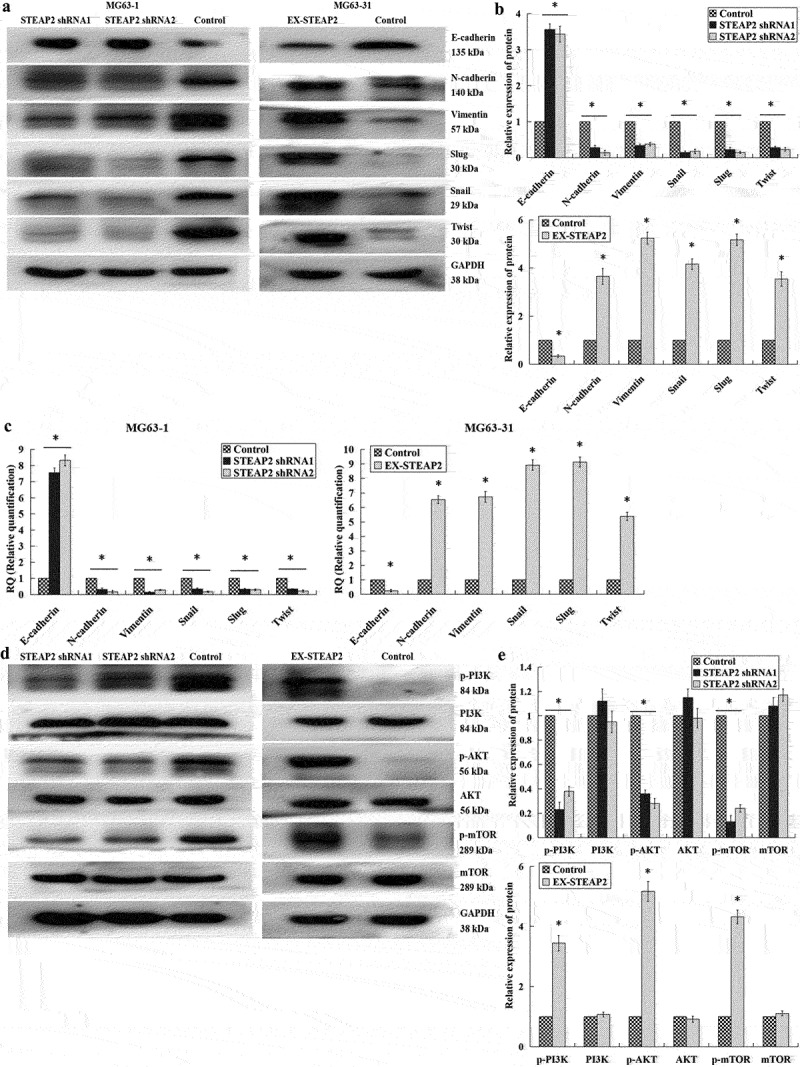
Effects of down-regulated or over-expressed STEAP2 on the key EMT hallmarks and the PI3K/Akt/mTOR pathway. According to the results of western-blot (a) and its relative gray value (b), as well as qRT-PCR outcomes (c), STEAP2 knockdown significantly increased the epithelial marker E-cadherin level and reduced the levels of N-cadherin and vimentin, the interstitial markers. Besides, the levels of transcriptional factors like Slug, Snail and Twist were also significantly reduced, indicating the EMT event repression by the STEAP2 down-regulation. STEAP2 up-regulation induced EMT, showing the E-cadherin level decline, as well as the elevation of N-cadherin, Vimentin, Slug, Snail and Twist levels. According to western-blot outcomes (d) and its relative gray value (e), the STEAP2 repression weakened the phosphorylation of PI3K, AKT, and mTOR pronouncedly, while the up-regulation of STEAP2 enhanced such phosphorylation prominently. *P < .05.

**Figure 6.**
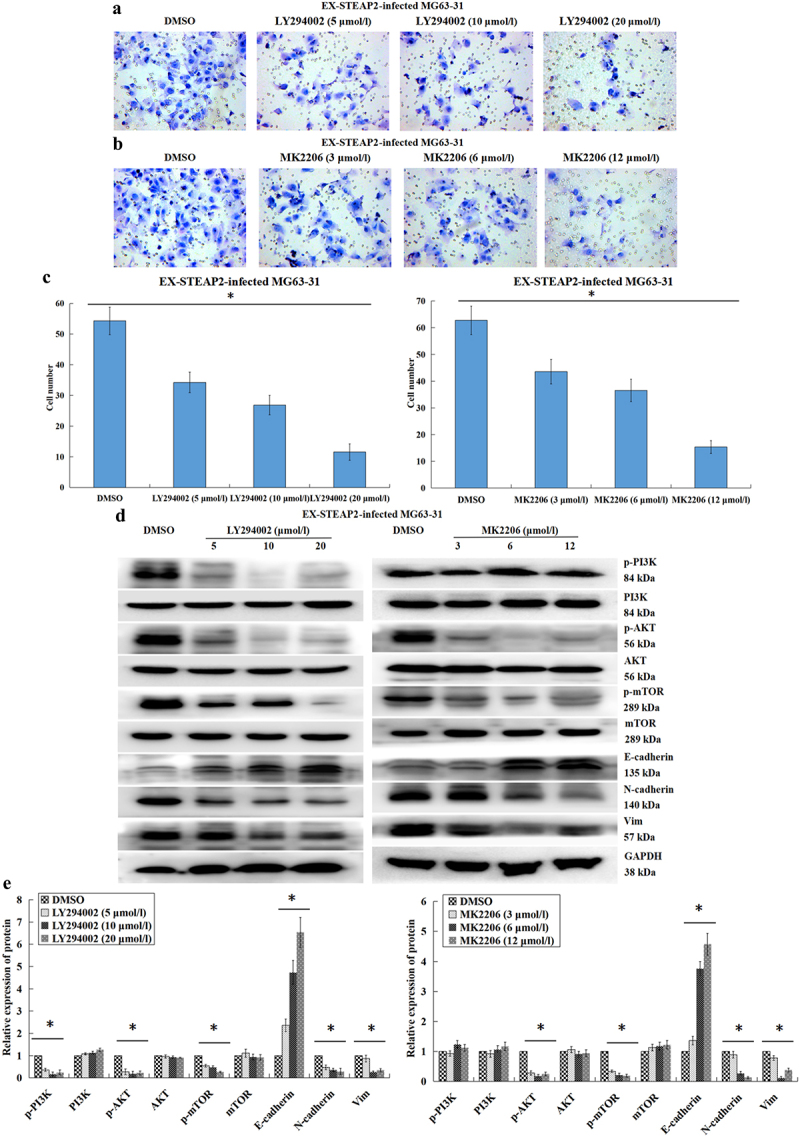
Effects of PI3K/Akt signaling pathway inhibitors on the osteosarcoma cell invasiveness and EMT progress. The cells infected with STEAP2 cDNA were treated using 5, 10 and 20 μmol/L PI3K inhibitor LY294002 and the AKT inhibitor MK2206 (3, 6, and 12 μmol/L) for 48 h. (AB) By transwell chamber invasion assay, the STEAP2 over-expression-mediated enhancement of the osteosarcoma cell invasive capacities was inhibited by both inhibitors. (c) The inhibitory effect was quantitatively dependent on and increased with the increase in the concentrations of the inhibitors. By western-blot (d) and its relative gray value (e), the PI3K signaling pathway activated by STEAP2 over-expression was also inhibited by both inhibitors, and with the increase in the concentration of the inhibitors, the PI3K inhibitor LY294002 weakened the phosphorylation of PI3K, AKT and mTOR, while the AKT inhibitor MK2206 decreased the phosphorylation levels of AKT and mTOR, and both inhibitors inhibited the EMT process in a dose-dependent manner, with the E-cadherin up-regulation and the N-cadherin and Vimentin down-regulation.

EMT is known to be critical to the malignant progression of tumors and is characterized by the movement of epithelial cells from the underlying basement membrane into the tumor microenvironment and their subsequent acquisition of the mesenchymal phenotype, resulting in increased migratory and invasive capacity.^25^ Here, we describe the crucial function of STEAP2 in osteosarcoma cell infiltration and migration, which in turn led us to speculate that the differential regulation of STEAP2 might affect the EMT process. This hypothesis was subsequently supported by the results of our western blot experiments (Figure 5 a and b), which revealed that inhibition of STEAP2 significantly increased epithelial marker E-cadherin expression while downregulating both N-cadherin and vimentin expression. Moreover, the expression of various transcription factors including Slug, Snail and Twist were significantly reduced, indicating that the downregulation of STEAP2 expression inhibits the EMT process. These outcomes were then validated when STEAP2 overexpression was shown to induce EMT, reducing E-cadherin expression and increasing N-cadherin, vimentin, Slug, Snail and Twist expression. Additional qRT-PCR analysis confirmed these observations at the transcript level (Figure 5c). When combined with our previous data, which suggests that EFEMP2 facilitates the infiltration and migration of osteosarcoma cells through its activation of the PI3K/Akt/mTOR signaling pathway,^24^ we suggest that STEAP2 is a likely effector in this process, acting as a critical conduit for proliferation, infiltration, and EMT. Given this, we hypothesized that STEAP2, similar to EFEMP2, likely exerts its function via activation of the PI3K/Akt/mTOR axis, which was then confirmed by western blot (Figure 5 d and e) with the downregulation of STEAP2 expression reducing PI3K, AKT and mTOR phosphorylation and the upregulation of STEAP2 expression significantly increasing the phosphorylation of these proteins. These observations were then confirmed as follows: cells infected with STEAP2 cDNA were treated with PI3K/AKT pathway inhibitors LY294002 (5, 10, and 20 μmol/L) and MK2206 (3, 6, and 12 μmol/L) for 48 h and evaluated using transwell chamber invasion assay. The assay results revealed that the introduction of the PI3K/AKT inhibitor reversed the enhanced invasiveness of these osteosarcoma cells (Figure 6 a and b) and that these effects were concentration dependent (Figure 6c), confirming the direct relationship between STEAP2 and the PI3K/AKT pathway. As indicated by subsequent western blot (Figure 6 d and e), their links to EMT were also confirmed, which revealed that both inhibitors inhibited the EMT process in a dose dependent manner, with increased E-cadherin and decreased N-cadherin and vimentin expression.

### EFEMP2 targets STEAP2 promoting EMT and activating the PI3K/Akt/mTOR pathway

**Figure 7.**
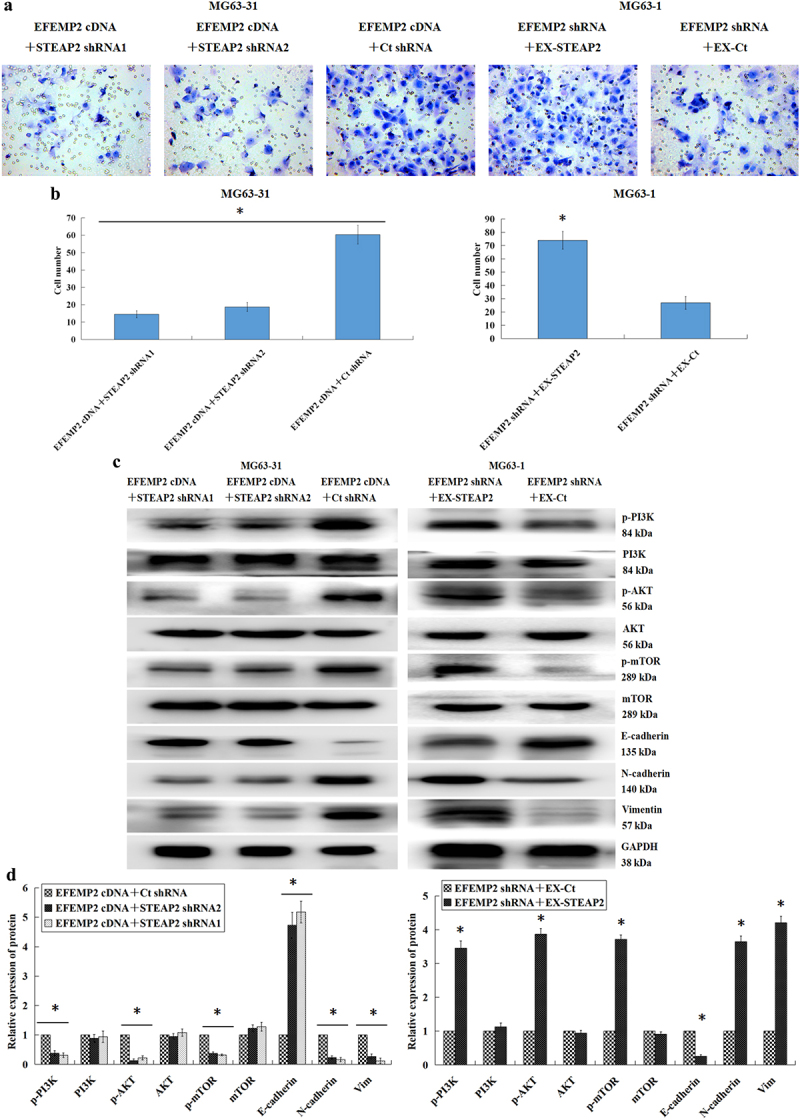
EFEMP2 targeted STEAP2 to promote the invasion of osteosarcoma cells, and activate EMT via the PI3K/Akt/mTOR pathway. (a) Transwell invasion assessment revealed that in osteosarcoma cells co-transfected using EFEMP2 cDNA and STEAP2 shRNA1 or STEAP2 shRNA2, down-regulated expression of STEAP2 significantly inhibited cell invasion enhanced by EFEMP2 over-expression, in contrast, in osteosarcoma cells co-transfected with EFEMP2 shRNA and STEAP2 cDNA, the over-expression of STEAP2 reversed the invasive ability of osteosarcoma cells inhibited by EFEMP2 down-regulation. (b) A significantly lower number of osteosarcoma cells co-transfected with EFEMP2 cDNA and STEAP2 shRNA1 or STEAP2 shRNA2 invaded Matrigel than those transfected with EFEMP2 cDNA alone. Osteosarcoma cells co-transfected with STEAP2 cDNA and EFEMP2 shRNA invaded Matrigel more markedly than cells transfected with EFEMP2 shRNA alone. By western-blot (c) and its relative gray value (d), STEAP2 knockdown suppressed the PI3K/Akt/mTOR axis and the EMT event, which were both activated by EFEMP2 over-expression, accompanied by the E-cadherin up-expression, the N-cadherin and vimentin down-expression, as well as the weakened phosphorylation of PI3K, AKT and mTOR. However, the exogenous over-expression of STEAP2 significantly enhanced the EMT event and initiated the PI3K/Akt/mTOR axis, both of which were repressed via the EFEMP2 down-regulation, accompanied by the E-cadherin down-expression, the N-cadherin and vimentin up-expression, as well as the enhanced phosphorylation of PI3K, AKT and mTOR. Magnification×200, *P < .05.

**Figure 8.**
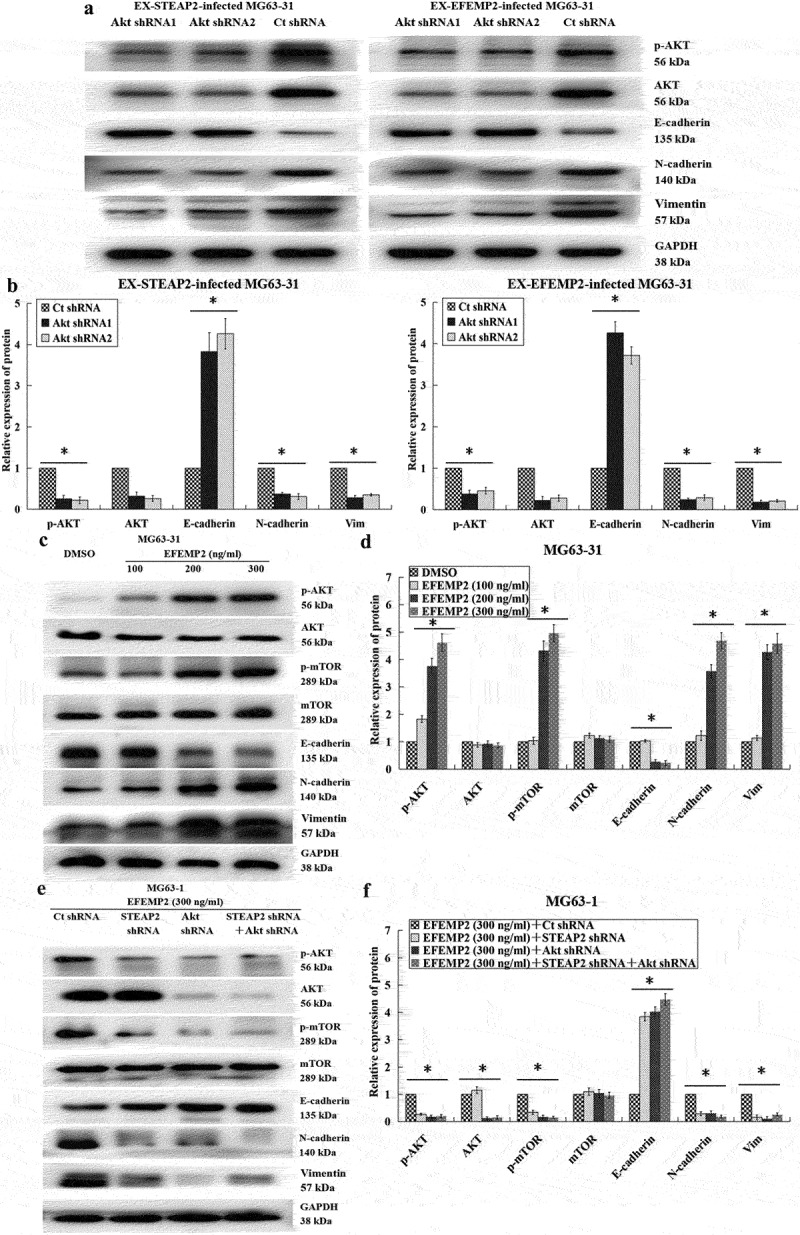
The significance of the PI3K/Akt/mTOR axis in the interactions of EFEMP2 with STEAP2 and its functions in EMT. By western-blot (a) and its relative gray value (b), the EMT process induced by EFEMP2 or STEAP2 over-expression was significantly inhibited after Akt down-regulation, and the pathway was inactivated, along with the E-cadherin up-expression, the N-cadherin and Vimentin down-expression, and reduced phosphorylation level of AKT. By western-blot (c) and its relative gray value (d), the lowly invasive osteosarcoma subclone MG63-31 cells were treated with purified EFEMP2 (100, 200, and 300 ng/ml) for 24 h. With the increase in EFEMP2 concentration, the expression of E-cadherin declined progressively, while the N-cadherin and vimentin levels were elevated progressively. Besides, there was elicitation of EMT, along with enhanced phosphorylation of AKT and mTOR. By western-blot (e) and its relative gray value (f), for osteosarcoma cells with the repression of either STEAP2 or Akt or both, purified EFEMP2 could not activate Akt pathway or EMT in the osteosarcoma cells. In other words, the down-regulated expression of STEAP2 or Akt blocked EFEMP2 from promoting the EMT process in osteosarcoma cells. *P < .05.

Given the above results, we further verified whether EFEMP2 directly targets STEAP2 to facilitate osteosarcoma infiltration and migration. EFEMP2 cDNA and STEAP2 shRNA1 or shRNA2 were co-transfected into less invasive MG63-31 cells, while highly invasive MG63-1 cells were co-transfected with EFEMP2 shRNA and STEAP2 cDNA. These treated cells were then evaluated using a transwell chamber invasion assay and western blot, which revealed that the downregulation of STEAP2 expression significantly inhibits the enhanced cellular invasion associated with EFEMP2 overexpression, while the overexpression of STEAP2 increased the invasive capacity of EFEMP2 deficient cells (Figure 7 a and b). In addition, we noted that STEAP2 knockdown repressed both EMT and PI3K/Akt/mTOR axis activation even in the presence of increased EFEMP2 expression, with the relative increase in E-cadherin expression and decrease in N-cadherin and vimentin expression, as well as the reduced phosphorylation of PI3K, AKT and mTOR in these cells. By contrast, exogenous overexpression of STEAP2 significantly enhanced EMT and PI3K/Akt/mTOR axis activation in cells with reduced EFEMP2 expression. These observations were supported by the fact that E-cadherin expression was downregulated; N-cadherin and vimentin expression was upregulated; and PI3K, AKT, and mTOR were significantly more phosphorylated in these cells (Figure 7 c and d). Thus, we can conclude that EFEMP2 facilitates EMT and initiates PI3K/Akt/mTOR axis activation, at least in part, via its direct interactions with STEAP2. Given this, we further clarified the significance of the PI3K/Akt/mTOR axis in the interactions of EFEMP2 with STEAP2 and its functions in EMT. This was accomplished by reducing Akt expression and then overexpressing EFEMP2 or STEAP2. These assays revealed that Akt inhibition reduced EMT even in the presence of EFEMP2 or STEAP2 and significantly reduced AKT phosphorylation (Figure 8 a and b). Based on these experimental results, we hypothesize that osteosarcoma cells secrete excessive EFEMP2, which in turn targets STEAP2 initiating the PI3K/Akt/mTOR axis and inducing EMT which in turn enhances osteosarcoma cell infiltration and migration. We validated this hypothesis by treating less invasive osteosarcoma MG63-31 cells with purified EFEMP2 (100, 200, and 300 ng/ml) for 24 h. The results revealed that increased EFEMP2 concentration reduced E-cadherin expression while increasing N-cadherin and vimentin expression, suggesting increased EMT. These cells were also characterized by increased AKT and mTOR phosphorylation (Figure 8 c and d) confirming our original hypothesis that EFEMP2 acts as a central regulator of this process. We then clarified the significance of STEAP2 and PI3K/Akt/mTOR axis by reducing STEAP2 and/or Akt expression in the EFEMP2-treated (300 ng/mL) cells. These experiments demonstrated that a reduction in either STEAP2 or Akt or both inhibited EMT and PI3K/Akt/mTOR axis activation in osteosarcoma cells even when treated with exogenous EFEMP2. Thus, downregulation of STEAP2 or Akt blocks EFEMP2-mediated induction of the EMT process in osteosarcoma cells (Figure 8 e and f). Taken together, these data suggest that EFEMP2 induces EMT and initiates the PI3K/Akt/mTOR axis partly via its interaction with STEAP2, thereby enhancing the migratory and invasive capacities of osteosarcoma cells and promoting the development of osteosarcoma.

## Discussion

Our previous study revealed that EFEMP2 facilitates osteosarcoma infiltration and migration via its activation of the PI3K/Akt/mTOR axis and that changes in EFEMP2 expression induce similar changes in STEAP2 expression. Here, we show that osteosarcoma tissues overexpress STEAP2 and that this is positively linked to poor clinical outcomes in these patients. STEAP2 is also overexpressed in highly invasive osteosarcoma and subclone cell lines, and similar to EFEMP2, it also induces EMT through the PI3K/AKT/mTOR axis. In addition, our data shows that the overexpression of this protein increases infiltration and migration and that EFEMP2 initiates the Akt pathway triggering EMT via its direct regulation of STEAP2, thereby intensifying the migratory and invasive capacities of osteosarcoma cells. We found that inhibition of STEAP2 or Akt expression via RNA interference prevents osteosarcoma cell invasion induced by the endogenous or exogenous overexpression of EFEMP2. Our data, thus, suggest that EFEMP2 partly targets STEAP2 to promote osteosarcoma progression by inducing EMT via the PI3K/AKT/mTOR axis.

As our findings suggest, high STEAP2 levels are closely related to increased histological grade, positive metastasis to lymph nodes and poor patient outcomes. In addition, knockdown of STEAP2 reduces the proliferation, clonogenesis, infiltration, and mobility of osteosarcoma cells, whereas STEAP2 upregulation promotes proliferation, clonogenesis, mobility, and invasion in these cells. While most research regarding the relationships between STEAP2 and cancerous tissues remains at the inception stage, some indicators are clearly available from its defined role in prostatic carcinoma progression. High levels of STEAP2 have been described in various prostatic carcinoma tissues, with this expression known to increase the malignant phenotype of these oncocytes.^14,26^ In addition, increased STEAP2 expression has been detected in the bone tissues of patients with metastatic prostate cancer.^27^ However, STEAP2 seems to act as a tumor suppressor gene in breast cancer, where its increased expression is positively correlated with improved prognosis.^28^ In addition, both *in vivo* and *in vitro* functionality tests reveal that STEAP2 reduces breast cancer cell proliferation and infiltration via the active repression of the PI3K pathway.^29^ Analysis of public lung cancer datasets reveals that STEAP2 expression is very low in tumor tissues and that its expression is closely associated with patients’ prognosis.^30^ Here, we found that STEAP2 is likely to act as an oncogene, playing a similar promotional role to EFEMP2 in osteosarcoma progression. To our knowledge, this is the first study to explore the relationship between STEAP2 and osteosarcoma, and similar to EFEMP2 expression, we found that STEAP2 was also highly expressed in osteosarcoma and likely to affect EMT through the PI3K/AKT/mTOR axis.

EMT is a key factor in oncocyte migration and invasion,^31^ and downregulation of STEAP2 represses EMT through reduced PI3K/AKT/mTOR axis activity, which in turn reduces osteosarcoma cell migration and infiltration. Moreover, upregulation of STEAP2 promotes EMT via activation of the PI3K/AKT/mTOR axis thereby enhancing the metastatic and invasive capabilities of osteosarcoma cells. In this study, we clearly show that changes in EFEMP2 expression directly affect STEAP2 expression in osteosarcoma. Give this, we hypothesized that EFEMP2 is closely related to STEAP2, and further experimental results revealed that knockdown of STEAP2 expression in EFEMP2 cDNA-transfected osteosarcoma cells significantly reduced the increased invasive capacity conferred by EFEMP2 overexpression. This was then validated by the fact that overexpression of STEAP2 in EFEMP2 shRNA-transfected osteosarcoma cells increases the invasive capacity of these cells even in the absence of EFEMP2. Moreover, overexpression of STEAP2 reversed the suppressive role of EFEMP2 inhibition on EMT and PI3K/AKT/mTOR axis activation. Similarly, repression of STEAP2 reversed the effect of EFEMP2 overexpression, and exogenous EFEMP2 activated the Akt pathway and EMT process in osteosarcoma cells but had no impact on cells with reduced STEAP2 or Akt expression. Taken together, these findings indicate that EFEMP2 likely regulates EMT and activates the PI3K/AKT/mTOR axis by targeting STEAP2, thereby facilitating osteosarcoma cell infiltration and migration. To date, there has been little investigation into the up- and downstream mechanisms controlling EFEMP2 and STEAP2 expression; however, this work suggests that these mechanisms may have a significant impact on the treatment of osteosarcoma. This work presents the first attempt to explore the effect of EFEMP2 on STEAP2.

In conclusion, STEAP2 is upregulated in osteosarcoma tissues and exhibits a positive correlation between its expression and the development of malignant osteosarcoma phenotypes and poor patient outcomes. In addition, similar to EFEMP2, STEAP2 may induce EMT through the PI3K/AKT/mTOR axis and facilitate osteosarcoma cell infiltration and migration. EFEMP2 is also likely to partly target STEAP2 to promote osteosarcoma progression. The effect of EFEMP2 was lost when STEAP2 was inhibited, and given the extensive roles of EFEMP2 and STEAP2 in tumor development, studies on the mechanisms underlying their interaction and regulation are necessary to facilitate the development of targeted drugs that can effectively inhibit invasion and metastasis.

## Supplementary Material

Supplemental Material

## Data Availability

All data generated or analyzed during this study are available from the corresponding author on reasonable request.
