# Supplementary figures and images for: STEAP2 promotes osteosarcoma progression by inducing epithelial–mesenchymal transition via the PI3K/AKT/mTOR signaling pathway and is regulated by EFEMP2
Source: Cancer Biol Ther. 2022 Oct 31;23(1):1–16. doi: 10.1080/15384047.2022.2136465 (PMC9629848; doi:10.1080/15384047.2022.2136465)

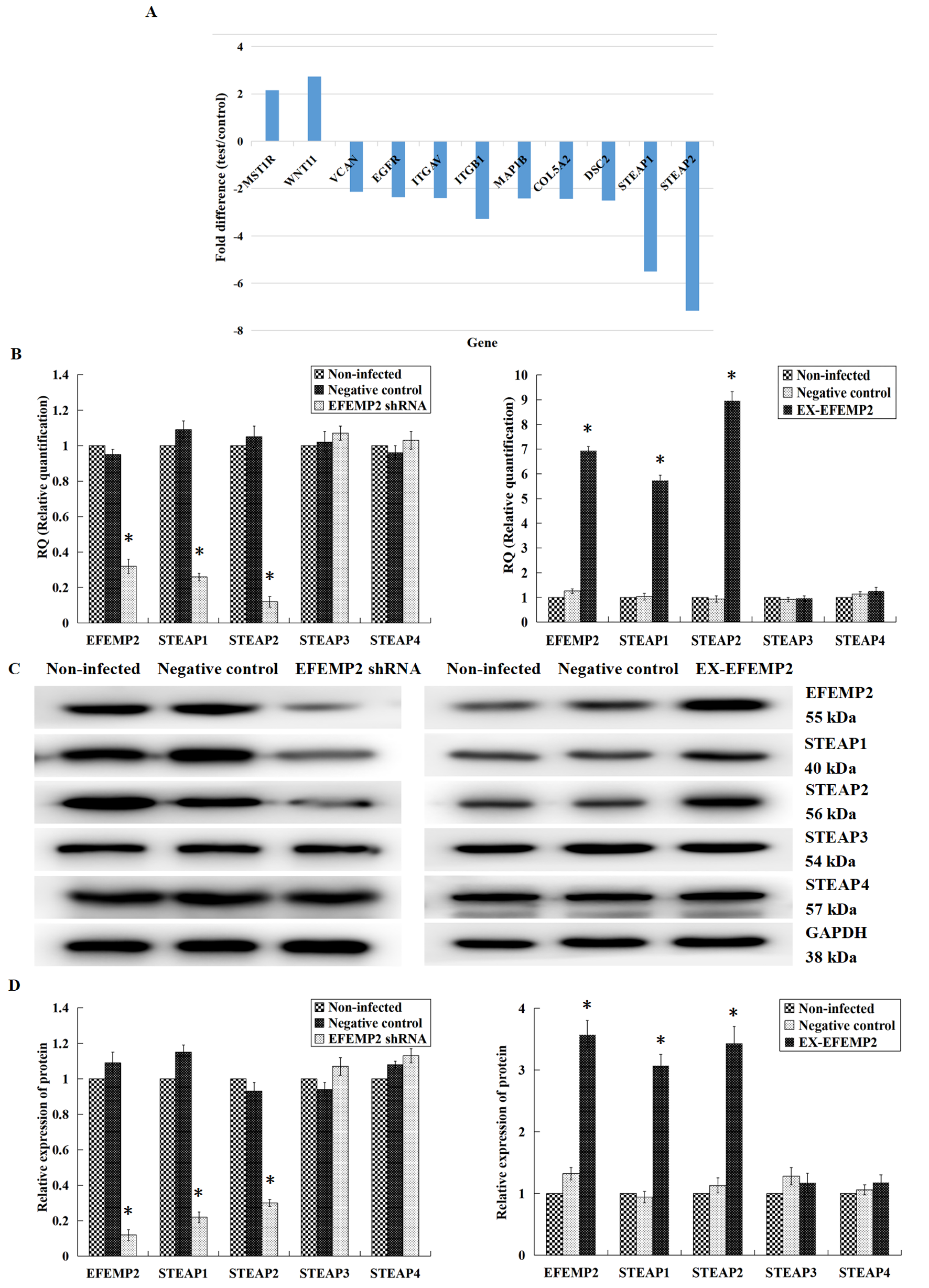

Supplement: Supplemental Material [file KCBT_A_2136465_SM2877.tif]
